# Path Tracing vs. Volume Rendering Technique in Post-Surgical Assessment of Bone Flap in Oncologic Head and Neck Reconstructive Surgery: A Preliminary Study

**DOI:** 10.3390/jimaging9020024

**Published:** 2023-01-20

**Authors:** Nicolò Cardobi, Riccardo Nocini, Gabriele Molteni, Vittorio Favero, Andrea Fior, Daniele Marchioni, Stefania Montemezzi, Mirko D’Onofrio

**Affiliations:** 1 Radiology Unit, Department of Pathology and Diagnostics, University Hospital of Verona, Piazzale Aristide Stefani, 1, 37126 Verona, Italy; 2 Otolaryngology-Head and Neck Surgery Department, University Hospital of Verona, Piazzale Aristide Stefani, 1, 37126 Verona, Italy; 3 Unit of Maxillo-Facial Surgery and Dentistry, University of Verona, P.le L.A. Scuro 10, 37134 Verona, Italy; 4 Department of Radiology, G.B. Rossi University Hospital, University of Verona, 37134 Verona, Italy

**Keywords:** volume rendering, path tracing, 3D reconstruction, maxillo-facial surgery

## Abstract

This study aims to compare a relatively novel three-dimensional rendering called Path Tracing (PT) to the Volume Rendering technique (VR) in the post-surgical assessment of head and neck oncologic surgery followed by bone flap reconstruction. This retrospective study included 39 oncologic patients who underwent head and neck surgery with free bone flap reconstructions. All exams were acquired using a 64 Multi-Detector CT (MDCT). PT and VR images were created on a dedicated workstation. Five readers, with different expertise in bone flap reconstructive surgery, independently reviewed the images (two radiologists, one head and neck surgeon and two otorhinolaryngologists, respectively). Every observer evaluated the images according to a 5-point Likert scale. The parameters assessed were image quality, anatomical accuracy, bone flap evaluation, and metal artefact. Mean and median values for all the parameters across the observer were calculated. The scores of both reconstruction methods were compared using a Wilcoxon matched-pairs signed rank test. Inter-reader agreement was calculated using Spearman’s rank correlation coefficient. PT was considered significantly superior to VR 3D reconstructions by all readers (*p* < 0.05). Inter-reader agreement was moderate to strong across four out of five readers. The agreement was stronger with PT images compared to VR images. In conclusion, PT reconstructions are significantly better than VR ones. Although they did not modify patient outcomes, they may improve the post-surgical evaluation of bone-free flap reconstructions following major head and neck surgery.

## 1. Introduction

Modern multi-detector computed tomography (MDCT) can acquire high-resolution images in a relatively low acquisition time [1,2]. In addition, the resulting isotropic millimetric or sub millimetric voxels allow multiplanar image reformatting without loss of spatial resolution. Although high quality, the acquired images and derived bi-dimensional reconstruction may not be as informative as a three-dimensional volume that radiologists and other physicians can rotate, zoom in and out or cut, to highlight or reveal structures of interest [3]. For these reasons, 3D reconstructions may be helpful to visualise complex anatomy clearly and synthetically. 

The Volume Rendering (VR) technique represents today the standard for 3D visualisation, transforming cross-sectional imaging into 3D images. This technique relies on a cast of a single simulated ray of light across the volume for each pixel image. The ray does not stop on the surface, and, going through the volume, it samples the volume data one way. Therefore, this technique does not consider the ray scatter or ray extinction once the ray hits the surface of the volume. To overcome this, a non-realistic shading model simulates the scatter effects, if needed [4]. 

Another recent approach to visualise 3D volume data is Path Tracing (PT) [5] or its similar commercial implementation, Cinematic Rendering (CR) [6]. In these rendering algorithms, the light model is far more complex. PT includes light scattering and extinction, simulating how the natural light ray behave. The resultant model has many light rays, most of them with complex paths and multiple reflections. The calculation of light scatters and extinctions is computationally expensive, so the light rays and reflections are usually artificially limited. Moreover, the lighting sources are very different compared to VR. There is a global illumination approach in PT or CR, while in VR, there are a finite number of synthetic light sources [7]. 

As a result, PT or CR images look more natural and physically accurate than VR reconstructions, with more realistic lighting and shadows, which leads to a better depth of field perception [8]. This kind of “photorealistic” appearance may be helpful in post-surgical imaging to better evaluate the immediate outcome of the surgery. 

The primary endpoint of this paper is to investigate if these reconstructions are superior to traditional VR images in evaluating complex post-surgical head and neck anatomy with bone flap reconstruction. 

## 2. Materials and Methods

### 2.1. Patients Selection

To investigate the hypothesis, a retrospective study of a consecutive series of patients who underwent head and neck reconstructive surgery comprehensive of bony reconstruction with free flaps was performed. Informed consent to perform CT scan was waived. Patients were treated in a tertiary center from 2017 to 2021 (a mean of 7.8 patients/year). Inclusion and exclusion criteria are listed in Table 1.

Post-traumatic bone reconstructions were excluded from this study to maintain the study group homogeneous from a surgical point of view, especially for bone flap evaluation. 

### 2.2. CT Acquisition and Post Elaboration

To assess the bone flap, we acquired a CT scan 24 h after surgery, as our head and neck surgeons routinely required. The rationale of the post-surgery CT is to check eventual bone flap and osteosynthesis material mispositioning that may benefit from immediate surgical correction. Fortunately, no patients required a revision of bone flap positioning after the post-surgery CT in our series. CT scans were acquired on 64 rows of multi-detector equipment (Philips Brilliance 64, Philips, Eindhoven, Nederland). Parameters were as follows: 120 kV, 200 mAS, slice thickness 0.8 mm, pitch 0.4, bone and soft tissue kernel reconstruction. 

For every patient, two VR and two PT 3D reconstructions, with the same angle of view, were elaborated on a workstation with an Nvidia RTX 2080 Ti graphic processing unit (GPU), respectively with a free dicom viewer (Medixant. RadiAnt DICOM Viewer. Version 2020.2. 19 July 2020. URL: https://www.radiantviewer.com, accessed on 28 August 2020) for VR and a custom MevisLab script (MeVisLab version 3.4, MeVis Medical Solutions AG) and his PathTracing module. All the anonymised images, in the form of screenshots, were arranged randomly in two separate files for VR and PT.

### 2.3. Images Evaluations and Statistical Analysis

The images were then evaluated independently by five observers, two radiologists with 2 and 10 years of expertise in the field (NC and MDO), one head and neck surgeon with 10 years of experience (VF) and two otorhinolaryngologists with 2 (RN) and 15 years (GM) of experience in bone flap reconstructions. If requested, the observers may require additional images to highlight the surgical reconstruction better. No observers required more projections. All the observers were blinded from the surgical procedure of the patients. Moreover, the head and neck surgeons and otorhinolaryngologists who evaluated the images did not perform the surgery to avoid patients’ recognition bias.

Every observer evaluated the images using a 5-point Likert scale reported in Table 2. For each parameter, mean and median values were calculated. A Wilcoxon matched-pairs signed-rank test was performed to compare the reconstructions. Interobserver agreement was assessed using Spearman’s rank correlation coefficient. A *p*-value of less than 0.05 indicated statistical significance for both tests. 

**Figure 1:**
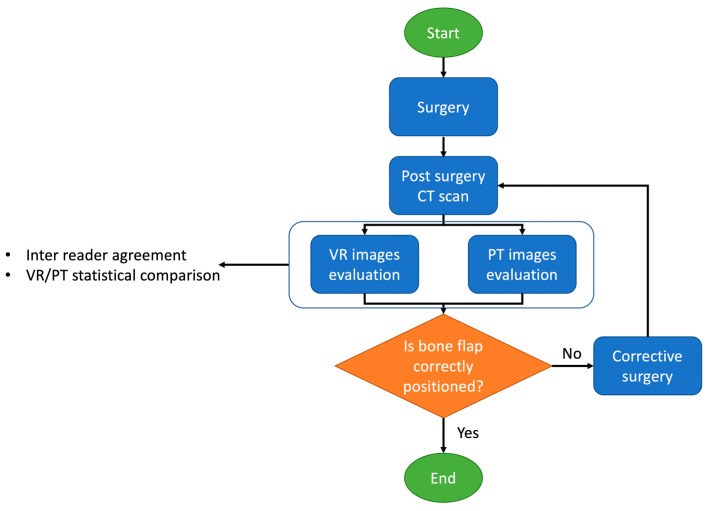
Flowchart of the study.

In Figure 1 is summarised the flowchart of the study.

## 3. Results

### 3.1. Patients

**Figure 2:**
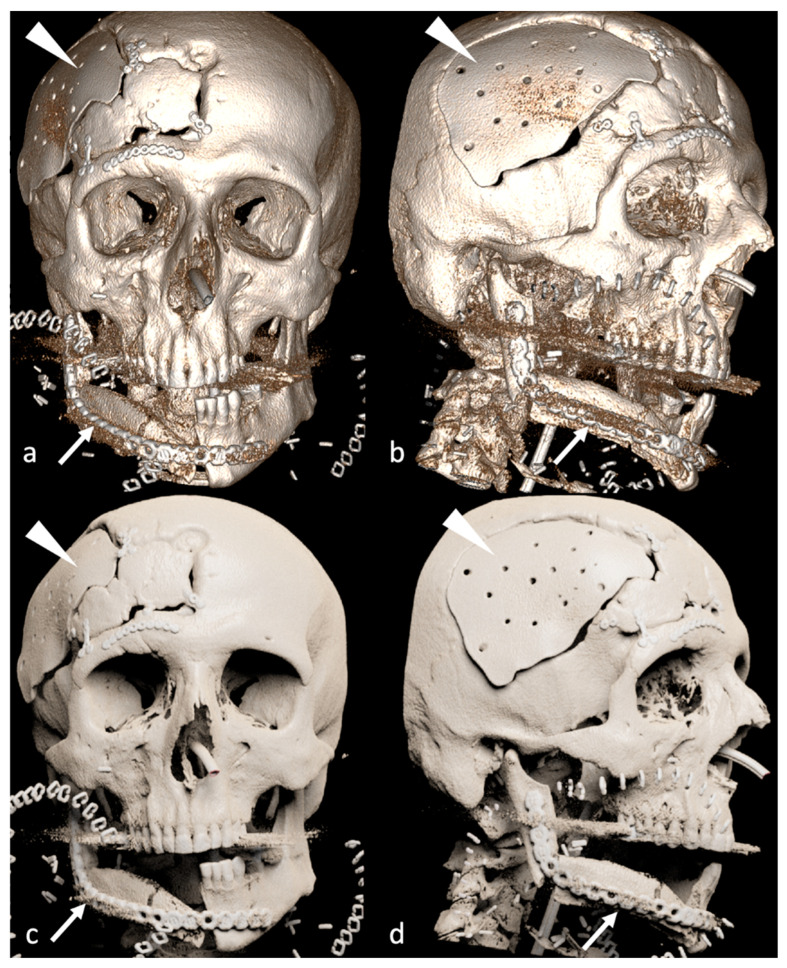
VR (**a**,**b**) and PT (**c**,**d**) reconstruction of scapular bone flap (arrows). The major depth of field and the better handling of metal artefacts of PT reconstructions are the key factors for better understanding post-surgery imaging compared to VR reconstructions. The patient underwent previous cranial vault surgery for trauma (arrowheads).

**Figure 3:**
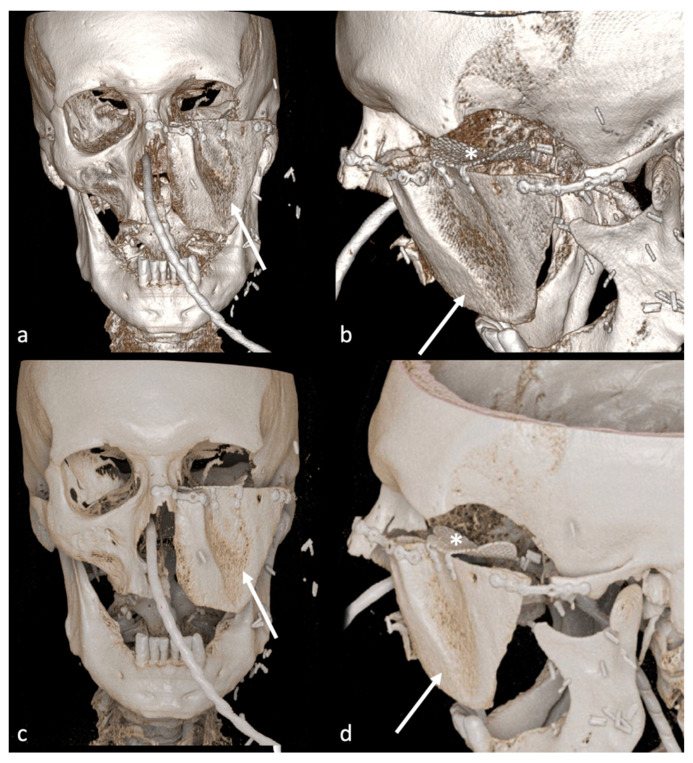
VR (**a**,**b**) and PT (**c**,**d**) reconstruction of the scapular bone flap (arrows). In PT image (**d**), the mesh of the orbit floor (asterisks) is better depictable than in VR image (**b**).

**Figure 4:**
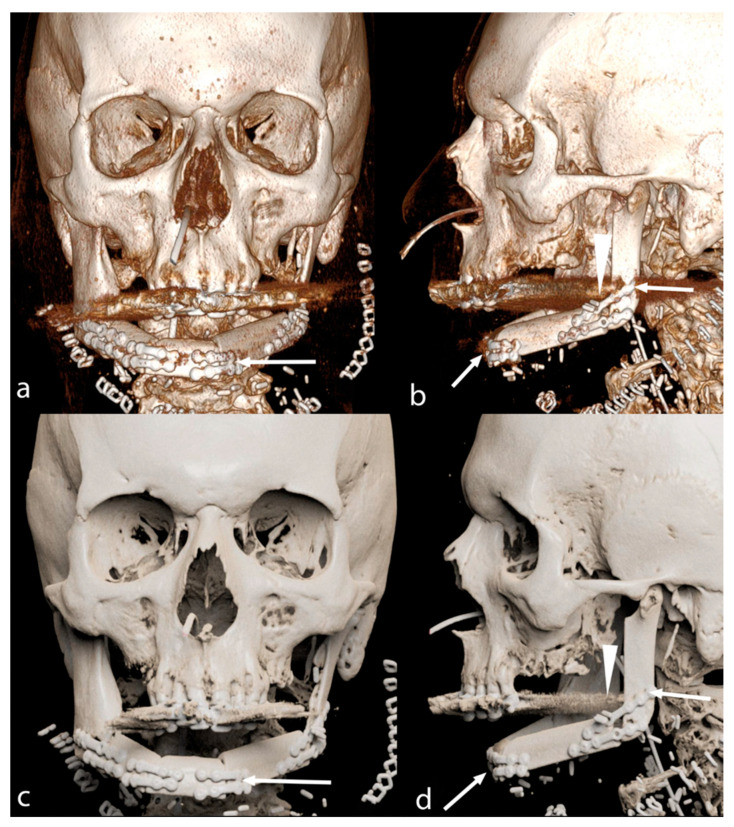
VR (**a**,**b**) and PT (**c**,**d**) reconstruction of fibular bone flap. In PT images, there are fewer metal artefacts from non-removable dental implants (arrowheads) and metal bone flap fixation materials (arrows).

According to the inclusion and exclusion criteria listed previously, 39 patients (25 males and 15 females; mean age 58.18 ± 12.10 years) were enrolled in the study. Among bone flap reconstructions, there were 10 scapular (Figure 2 and Figure 3), 28 fibular (Figure 4) and one medial femoral condyle flap. There were 25 squamous cell carcinoma patients (19 primary and six recurrent. According to TNM classification, there were 23 T4 and two T3 tumors), three ameloblastomas, one ameloblastic carcinoma (T4), one poorly differentiated carcinoma (T4), one basal cell carcinoma (T4), one osteosarcoma (T2 according to Enneking classification), one metastasis from breast adenocarcinoma, one inverted papilloma, one myxoid haemangioma, one radio-induced osteoblastic osteosarcoma, two bone radio-necrosis and one osteomyelitis.

### 3.2. Image Analysis—VR vs. PT

PT images were considered significantly superior to VR 3D reconstructions by all readers (*p* < 0.05), with similar evaluation across all the four parameters considered, except for metal artefacts for reader RN, which didn’t find statistically significant differences between VR and PT images (Table 3). 

Similar results were found if all readers’ median and mean values were considered together. In particular, the general image quality had a median and mean score of 4.00 and 3.99 in PT compared to 3.00 and 3.17 in VR reconstructions. PT images retained higher anatomical accuracy (4.25 and 4.09 median and mean value, respectively), than VR images. The bone flap was better depicted in PT images, with a median and mean score of 4.25 and 4.11, compared to 3.25 and 3.11 in VR images. Furthermore, PT reconstructions are less prone to metal artefacts than VR reconstructions, with a median and mean score of 3.75 and 3.69 (Table 4).

### 3.3. Image Analysis—Inter-Reader Agreement

**Figure 5:**
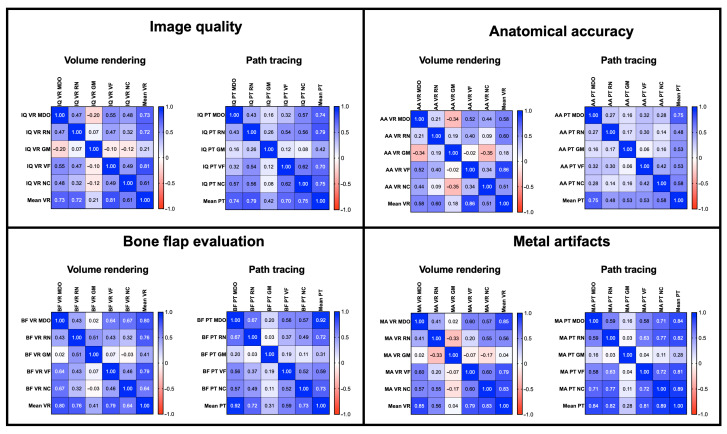
Inter-reader agreement correlation matrixes across all four parameters in VR and PT reconstructions (red negative correlation, blue positive correlation).

The inter-reader agreement was moderate to strong across three readers with a maximum agreement for image quality of ρ = 0.55 (VR) and 0.62 (PT), for anatomical accuracy of ρ = 0.518 (VR) and 0.42 (PT), for bone flap evaluation of ρ = 0.67 (VR) and 0.67 (PT), for metal artefacts of ρ = 0.60 (VR) and ρ = 0.77 (PT) (Figure 5). As shown in Table 3, despite the fourth reader (GM) finding PT significantly better than VR images, he generally showed no significant agreement with the other observers. 

However, there is a tendency for a better agreement in path tracing than volume rendering images among all readers.

## 4. Discussion

In this paper, we quantitatively investigated the possible superiority of PT compared to VR reconstructions in complex head and neck surgery followed by free bone flap reconstructions. The superiority of PT images resulted statistically significant across the different readers. Moreover, PT reconstructions are rated to be less prone to metal artefacts by three out of five readers (Figure 3), which may help further the evaluations of the bone flap in case of a non-removable dental prosthesis or metal fixation materials used in the surgery. These advantages may lead to a better post-surgical evaluation of bone flap reconstruction and immediate surgical revision if something is not correctly positioned. However, in our series, none of the patients required surgical revision even if evaluated only with VR reconstructions, so the clinical impact of these images remains unclear. 

Despite the readers finding that almost all parameters were better defined in PT than in VR images, the interrater agreement was low to moderate and non-significant for one reader (GM), who still judged PT images significantly better than VR images. Looking at the single scores assigned by readers, this may be due to a slightly different evaluation among the same patients. However, as shown in Figure 3, the interrater agreement in PT images is generally higher than in VR rendering images, probably because of consistently higher scores of PT reconstructions. 

Traditional VR 3D reconstructions have already proved helpful in evaluating complex anatomy in pre-and post-surgical settings in otolaryngology [9], craniofacial surgery planning [10,11], in trauma patients [12,13] and vascular pathology [14]. 

On behalf of volumetric visualisation, PT rendering is different from VR to generate 3D images. The newest technique allows elaborating photorealistic representation of volumetric data with a more natural shape, shadows and depth of field. PT, also called Cinematic Rendering (CR), has already been demonstrated to be feasible in multiple clinical situations, such as gastro-intestinal [15,16,17,18,19], genito-urinary [20,21,22], cardiovascular [23,24,25] and musculoskeletal imaging [26]. However, most papers on PT or CR usually showcase the technique without evaluating the benefits of these kinds of images generated. 

In literature, few papers quantitatively investigate this aspect, as conducted in this paper. Binder et al. evaluated the role of CR compared to conventional computed tomography in anatomy teaching. They found an overall time reduction of image interpretation by medical students of about 65.56%. Moreover, musculoskeletal and vascular anatomy rendering was significantly rated higher than conventional CT visualisation [27]. Vascular anatomy for pre-surgical planning was also significantly better evaluated and with faster comprehension with CR [28]. However, CR was found inferior to axial contrast-enhanced angiography in predicting vascular invasion by deep soft tissue sarcomas, so these images should be used in association with traditional 2D visualisation [29]. 

Wollschlaeger et al. evaluated the impact of CR compared to VR for pre-surgical lower limb fracture. They conclude that CR images are significantly superior to VR in anatomical accuracy and image quality [30]. 

This technique’s “photorealistic” nature of the images generated may better comprehend complex situations. The different and more complex lighting models used and, consequently, the realistic shadows of the images highlight depth perception. This is especially evident in overlapping anatomical structures, like maxillo-facial anatomy. Steffen et al. recently found this in applying Cinematic Rendering to facial skeleton [31]. Moreover Elshafei et al. demonstrated that CR leads to a significantly faster and easier comprehension of surgical hepatopancreatobiliary tumors anatomy. More interestingly, the results are independent of surgeon experience level [28]. 

One drawback of these reconstructions is that they require more computational power than traditional VR acquisition. For example, in our workstation, while interactive VR was rendered in real-time above 60 frames per second, a single PT image required about 5 s to generate, so it is impossible to visualise a PT volume in real-time like VR reconstructions to date. 

The study has several limitations. First of all, it is a retrospective study. A prospective clinical study will be more appropriate to evaluate the impact of the PT images compared to VR ones, and to evaluate if PT images lead to different post-surgical management of the patients. As mentioned previously, even with only the VR reconstructions, our head and neck surgeons did not perform corrective surgery on this series of patients. The second limitation is the relatively low number of cases. A higher number of patients may highlight the differences between the two techniques and modify the inter-reader agreement. Third, since generating PT images required elaboration time, the readers evaluated the patients based on a few VR and PT reconstructions pictures. This approach lacks interactivity granted by traditional volumetric visualization, hence may influence observers’ evaluation. However, all the observers did not require more images. Moreover, the paper focused on the differences in 3D imaging visualization technique, highlighted in the same images from the same point of view. The availability of a full 3D model may modify the score and need to be investigated once the PT rendering performance will be similar of VR to experience the same level of interactivity. The lower performance and the interface lag of PT rendering may be frustrating for some users.

## 5. Future Perspectives

Once established in a prospective study the impact of these images elaborated with the PT algorithm, a future perspective may be the application of virtual reality and high-performing computing. The combination may allow real-time visualisation of all acquired volume with enhanced depth of field perception and more visualising freedom.

## 6. Conclusions

Thanks to its enhanced lighting model with realistic shadows and better depth perceptions, PT images are superior to traditional VR images in general image quality, anatomical accuracy and bone flap evaluation. Moreover, three out of four readers rated PT images less prone to metal artefacts. Consequently, PT images may be more helpful than traditional VR images in comprehending post-surgery head and neck bone flap reconstructions. 

## Figures and Tables

**Table 1:** Inclusion and exclusion criteria.

Inclusion Criteria	Exclusion Criteria
- Major head and neck surgery and microvascular bone reconstruction for oncologic disease and/or its complications	- Absence of postoperative CT scan - Severe motion artefacts - Non-oncologic bone reconstruction

**Table 2:** Evaluated parameters and explanation of the 5-point Likert scale.

	Likert’s Scale Scores				
Parameters	1	2	3	4	5
Image Quality	Non-diagnostic	Poor quality	Good quality	Very good quality	Excellent quality
Anatomical Accuracy	Non-diagnostic	Poorly demarcated bone margins	Good demarcation of bone margins	Very good demarcation of bone margins	Excellent demarcation of bone margins
Bone Flap Evaluation	Non-diagnostic	Poor quality	Good quality	Very good quality	Excellent quality
Metal Artifact	Severe	Strong	Moderate	Mild	No artefacts

**Table 3:** Likert’s scale evaluation results across all readers and parameters. Values are expressed as median (MDO, RN, VF, GM, NC = readers; VR = volume rendering; PT = path tracing).

		Image Quality			Anatomical Accuracy			Bone Flap Evaluation			Metal Artifacts		
		VR	PT	*p*	VR	PT	*p*	VR	PT	*p*	VR	PT	*p*
**Reader**	**MDO**	3	4	<0.05	3	4	<0.05	2	4	<0.05	3	4	<0.05
	**RN**	3	4	<0.05	3	4	<0.05	3	4	<0.05	3	3	0.65
	**VF**	4	4	<0.05	4	5	<0.05	4	5	<0.05	3	4	<0.05
	**GM**	3	4	<0.05	3	4	<0.05	3	4	<0.05	3	4	<0.05
	**NC**	3	4	< 0.05	4	4	<0.05	3	4	< 0.05	3	4	<0.05

**Table 4:** Results of the Likert’s scale evaluation of ll readers considered together (VR = volume rendering; PT = path tracing; SD = standard deviation).

	Image Quality		Anatomical Accuracy		Bone Flap Evaluation		Metal Artefacts	
	VR	PT	VR	PT	VR	PT	VR	PT
**Median**	3	4	3	4	3	4	3	4
**Mean±SD**	3.19 ± 0.47	4.01 ± 0.56	3.24 ± 0.37	4.14 ± 0.45	3.19 ± 0.55	4.16 ± 0.56	3.15 ± 0.58	3.79 ± 0.64
***p***	<0.05		<0.05		<0.05		<0.05	

## Data Availability

Data is not available due to privacy restrictions.
